# Precise diagnosis of *Neisseria macacae* infective endocarditis assisted by nanopore sequencing

**DOI:** 10.1080/22221751.2020.1807411

**Published:** 2020-08-24

**Authors:** Jing-Wen Ai, Hong Liu, Hui-Xia Li, Qing-Xia Ling, Yan-Qin Ai, Si-Jia Sun, Xuan Wang, Bing-Yan Zhang, Jian-Ming Zheng, Jia-Lin Jin, Wen-Hong Zhang

**Affiliations:** a Department of Infectious Diseases, Huashan Hospital, Fudan University, Shanghai, People’s Republic of China; b Department of Clinical Laboratory, Huashan Hospital, Fudan University, Shanghai, People’s Republic of China; c Department of Infectious Diseases, No. 988. Hospital of Liberation Army, Henan, People’s Republic of China; d Department of Infectious Diseases, Qingpu Branch of Zhongshan Hospital, Fudan University, Shanghai, People’s Republic of China; e Department of Infectious Diseases, Xuchang Central Hospital affiliated to Henan University of Science and Technology, Henan, People’s Republic of China; f Department of Gastroenterology, Huashan Hospital, Fudan University, Shanghai, People’s Republic of China

**Keywords:** *Neisseria macacae*, *Neisseria* sp, Infective endocarditis, nanopore sequencing, valve replacement

## Abstract

Infective endocarditis caused by *Neisseria macacae* in humans is extremely rare. We presented here a case of *N. macacae* infective endocarditis in a 61-year-old man with a native aortic valve infection. *N. macacae* was isolated from blood culture and was detected by nanopore-based metagenomic sequencing in the vegetations. Finally, the patient recovered completely after surgery and antibiotic therapy.

## Introduction

Infective endocarditis (IE) caused by *Neisseria macacae* (*N. macacae*) in humans is extremely rare. The first case of *N. macacae* infective endocarditis in humans was reported in 2017 [1]. Unfortunately, that patient died 4 days after surgery in France [1]. Now, we present in this study a survival case of *N. macacae* infective endocarditis. The patient received cardiac vegetations surgical removal and underwent routine laboratory examination during the hospitalization. The blood culture reported *N. macacae*, while the vegetation culture was negative, and this does not fit the pathologic diagnostic criteria of the infective endocarditis, in which microorganisms should be identified by culture or histologic examination of a vegetation [2]. In order to further identify the possible causative pathogen in the vegetation, we performed high throughput sequencing on the vegetation tissues, which could provide a rapid, clinically non-biased actionable diagnosis method [3]. The nanopore sequencing of the vegetations confirmed that the detection of *Neisseria* sequences, a rare pathogen of infective endocarditis and qPCR validated the result in the end.

## Materials and methods

### Medical records and samples collection

Medical records of this patient were collected. After an aortic valve replacement, we performed culture and nanopore sequencing on the vegetations for culture and further detection with the consent of the patient. Informed consent was obtained from the patient for publication of his medical records and the vegetations after operation for further detection.

### Sequencing analysis

Total DNA was extracted from the vegetations using the TIANAmp Micro DNA Kit (TIANAmp; Tiangen Biotech, Tiangen, China) according to the manufacturer’s recommendations. After synthesizing second-strand DNA, DNA libraries were constructed by DNA fragmentation, end repair, A-tail addition, adapter ligation and PCR amplification. An Agilent 2100 Bioanalyser (Agilent, Santa Clara, CA, USA) was used for quality control of the DNA libraries. A sequencing library of the pathogen was prepared as described previously for GridION nanopore (Oxford, UK) sequencing [4]. Briefly, 300 ng of 1.2 × AMPure XP bead washed DNA of each sample was eluted in 7.5 μl of nuclease-free water, then gently mixed with 2.5 μl of unique barcoded tags separately for the tagmentation/fragmentation reaction, where DNA was incubated at 30°C for 1 min and at 80°C for 1 min. When multiplexing, DNA were pooled together in equal concentrations, then subjected to a 0.6 × AMPure XP bead wash and eluted in 10 μl of the buffer recommended in the manufacturer’s instructions (10 μl of 50 mM NaCl, 10 mM Tris HCl pH 8.0). Sequencing was performed on the GridION platform using R9.4.1 flow cells. The library (300–600 ng) was loaded onto the flow cell according to the manufacturer’s instructions. ONT MinKNOW software (v.1.4–1.13.1) was used to collect raw sequencing data, and ONT Albacore (v.1.2.2–2.1.10) was used for local base-calling of the raw data after sequencing runs were completed. The GridION was run for up to 48 h with WIMP/ARMA analysis performed on the first six folders (∼24,000 reads) for all samples. The first 2 h of data for all samples were analysed and recorded. The average depth for Nanopore is ∼400 base pairs (bp) and the error rate is ∼15%. Unicycling was carried out to combine the short Illumina reads and long Nanopore reads.

### Bioinformatics analysis and pathogen identification

Sequenced reads were first demultiplexed using home-cooked scripts, which have slightly higher accuracy than ONT’s recommended tool Guppy (2.3.5). Read length and mean quality score were then used to filter short reads (read length ≤500 nt) and low quality reads (mean q-score ≤8). Subsequently, host reads that could be aligned to human reference genome (GRCh38) were eliminated. After host depletion, reads were assigned to taxonomy by software centrifuge (version 1.0.4) and confirmed by megablast (2.7.1). Any species that have more than 2 reads and abundance over 1% would be considered as an identification.

### qPCR assays

qPCR assays were performed on a QuantStudio™ 5 Real-Time PCR System (Applied Biosystems™, LSA28139). The master mix of probe-based reactions consisted of 7.5 μl SGExcel GoldStar TaqMan Master (Sangon, B532932), 0.4 μl each of reverse and forward primers (final concentration 0.2 μM) and 0.4 μl of probe (final concentration 0.2 μM). For all SYBR Green-based qPCR: the master mix of SYBR Green-based reactions consisted of 7.5 μl of FastStart Universal SYBR Green Master (Roche, 4913850001), 0.4 μl each of reverse and forward primer (final concentration 0.2 μM). For one reaction, 2 μl of DNA template and nuclease-free water up to a total volume of 15 μl were added. The qPCR conditions were: pre-incubation at 95°C for 5 min, amplification for 40 cycles at 95°C for 30 s, 55°C for 30 s and 72°C for 30 s, with a final extension at 72°C for 5 min. Melt curve analysis (for SYBR Green qPCR) was performed at 95°C for 5 s, 65°C for 1 min, ramping to 95°C at 0.03°C s^–1^ in continuous acquisition mode, followed by cooling to 37°C. All probe-based confirmatory qPCR used the following conditions: pre-incubation at 95°C for 15 min, amplification for 40 cycles at 94°C for 15 s and 60°C for 1 min.

## Results

### Case presenting

**Figure 1.**
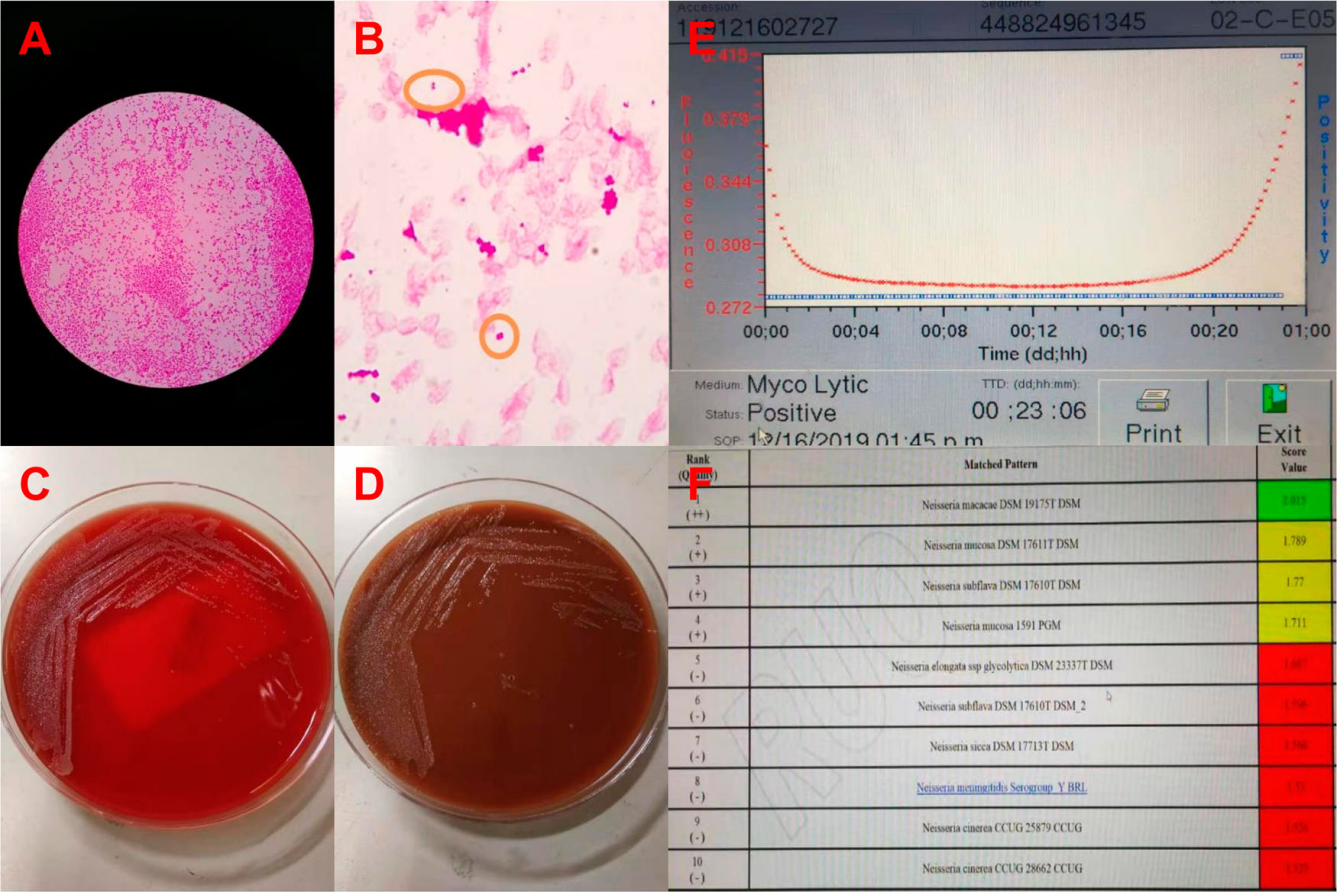
The blood culture was positive for *N. macacae* in 24 h. (A) Bacterial colony smear (Gram staining). (B) Blood culture was positive, then direct smear (Gram staining). (C) Blood plate for 72 h. (D) Chocolate plate for 72 h. (E) Time curve of blood bottle positive report. The blood culture was positive at 23 h 6 min. (F) The strain from blood culture was identified using MALDITOF MS.

In December 2019, a 61-year-old man was admitted to our hospital. He had been suffering from continuous fever for 4 weeks. He underwent pneumothorax operation in 1998 and underwent left renal space occupying surgery in 2009, which the pathological examination reported leiomyoma. He had more than 10 years of history of diabetes and no other known immune deficiency. In September 2019, he travelled to Jiuhua Mountain, where monkeys can be frequently seen along the way. During the physical examinations, a systolic heart murmur at the aortic area was heard. Laboratory findings showed white blood cell count was 8.53 × 10^9^/ml, the neutrophil count was 7.67 × 10^9^/ml (N89.9%) in the blood routine test, and C-reactive protein = 72 mg/L. The patient was diagnosed with suspect infective endocarditis and received penicillin soon after admission. Then, the trans-thoracic echography (TTE) showed aortic stenosis with moderate-to-severe aortic regurgitation and multiple aortic vegetations (vegetations can be seen on all three leaflets, and the larger ones were 8 × 5 mm on left lobe and 7 × 4 mm on right lobe). Due to the clear observation of the vegetation thought TTE, the transoesophageal echocardiogram was not further conducted in this patient. Two blood cultures collected on the outpatient clinics and the first day in hospital were positive for *N. macacae* at 23 h 6 min after sample collection (Figure 1A–E). Combined with the patient's clinical manifestations and laboratory tests, we diagnosed the patient as rare case of *N. macacae* infective endocarditis and the patient was continuously treated with intravenous penicillin. On the next day, the patient’s body temperature returned to normal and the third blood culture collected on the second day of hospitalization returned negative. An aortic valve replacement was performed after 1 week antibiotics therapy in another hospital so that the family members can take care of him conveniently. The vegetation culture was negative while further nanopore sequencing of vegetations and qPCR identified *Neisseria* sp. The patient continued to undergo antibiotic therapy for 6 weeks after surgery. Finally, the patient recovered completely.

### Identification of bacteria from blood culture

**Figure 2.**
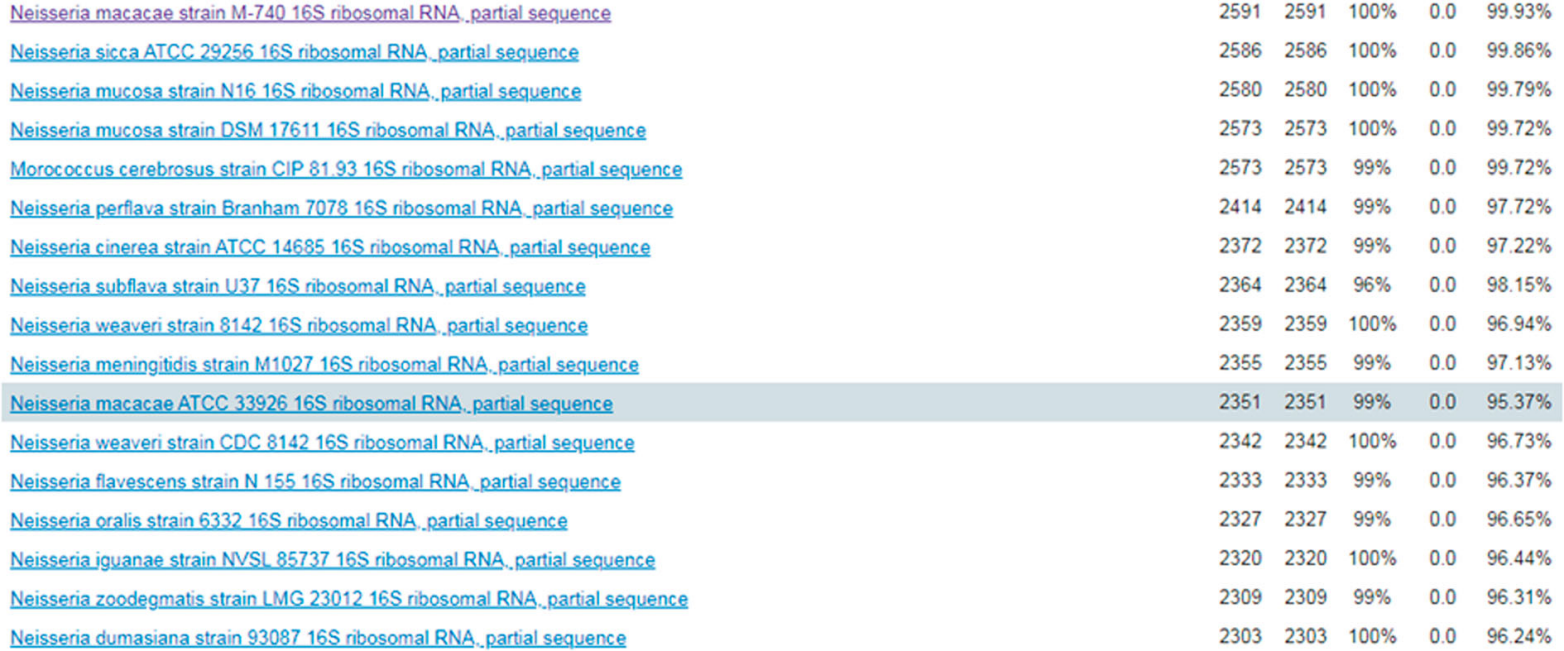
The results of 16S RNA sequencing. It was 99.93% of similarity with the genbank sequence reference: *N. macacae* strain M-740 16S ribosomal RNA part sequence.

The strain from blood culture was identified using matrix-assisted laser desorption ionization-time of fight mass spectrometry (MALDITOF MS, Bruker, Germany) in our laboratory. The strain best matched to *N. macacae* DSM 19175 T DSM, rank quality 1(++), score value 2.015 (Figure 1F). Furthermore, completed by 16S RNA sequencing of this organism, it was 99.93% of similarity with the genbank sequence reference: *N. macacae* strain M-740 16S ribosomal RNA part sequence (Figure 2).

### Sequencing analysis of vegetations

**Figure 3.**
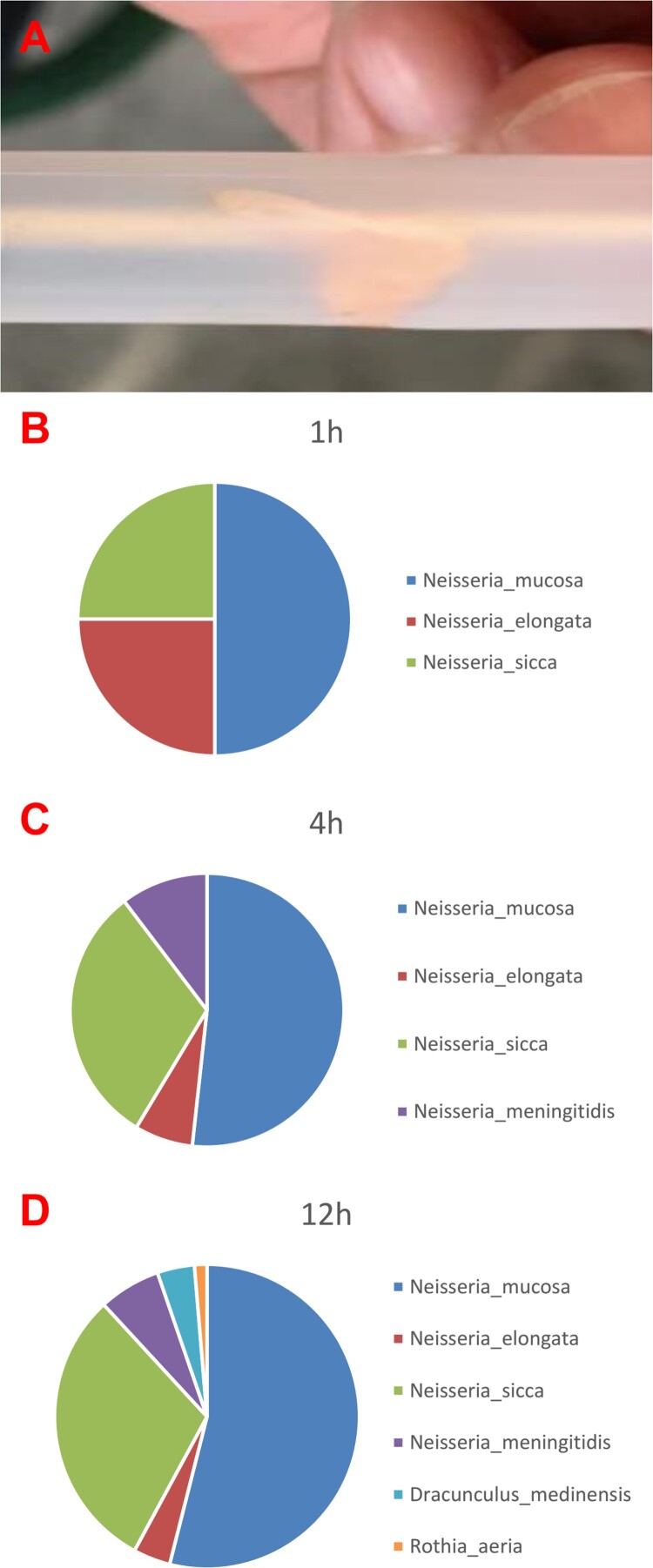
The results of vegetation detection. (A) Vegetations in sterile tubes, about 10 × 5 mm. (B) Sequencing analysis in 1 h. (C) Sequencing analysis in 4 h. (D) Sequencing analysis in 12 h.

A part of the vegetations was collected in sterile tubes after an aortic valve replacement for further high throughput sequencing detection (Figure 3A). Nanopore sequencing on the valve tissue in 1 h, 4 h and 12 h all identified the presence of *Neisseria* sp. The earliest positive result was reported at 1 h after the initiation of the sequencing and showed 4 reads of *Neisseria mucosa* (50%), 2 reads of *Neisseria elongata* (25%) and 2 reads of *Neisseria sicca* (25%). At 4 h, 15 reads *of Neisseria mucosa* (51.72%), 2 reads of *Neisseria elongate* (6.9%), 9 reads of *Neisseria sicca* (31.03%) and 3 reads of *Neisseria meningitidis* (10.34%) were reported. And 41 reads *of Neisseria mucosa* (53.95%), 3 reads of *Neisseria elongata* (3.95%), 23 reads of *Neisseria sicca* (30.26%), 5 reads of *Neisseria meningitidis* (6.58%), 3 reads of *Dracunculus medinensis* (3.95%) and 1 read of *Rothia aeria* (1.32%) were finally detected at 12 h, respectively (Figure 3B–D). *Neisseria mucosa* was the highest matched microorganisms detected in vegetation using nanopore sequencing. However, the genomic sequence of *N. macacae* was not in the DNA libraries, and *N. mucosa* was more than 99% of similarity with the *N. macacae* in genomic sequences. So, it was confirmed by specific qPCR assays leading to the identification of *N. macacae* (see Supplementary material).

## Discussion

Most of the *Neisseria* sp. genus are opportunistic pathogens, but two species, *N. meningitidis* and *N. gonorrhoeae*, are important pathogens in humans [1]. The most common pathogen among *Neisseria* species causing IE remain to be *N. gonorrhoeae*, usually presented with large vegetation that involves the aortic valve, accompanied by ring abscesses in young males. *N. elongate* infection is another aggressive form of endocarditis, many of which required valve replacement at early time [5–7]. *N. macacae* is a Gram-negative diplococcus and was first detected as a new species in the oropharynx of healthy Rhesus Monkeys [1]. To our knowledge, *N. macacae* is an uncommon cause of endocarditis, only one fatal case has been reported before our study. The patient of that fatal case underwent cardiac surgery on an emergency basis 5 weeks after the diagnosis of IE and died 4 days after surgery [1]. Our patient underwent cardiac surgery on 1 week after the diagnosis of IE, and thus, early surgery may be one of the key factors in decreasing the risk of systemic embolism and enhance survival. In this case, we further sequenced the vegetation by nanopore sequencing and identified the *Neisseria* sequences was detected in the vegetation, which further confirmed the diagnosis of *Neisseria* sp. infectious endocarditis.

Next-generation sequencing enabled rapid and accurate microbiological diagnostics could provide a clinically actionable diagnosis of uncommon pathogen and facilitate tailored treatments [8]. High throughput sequencing technologies could also help overcome some limitations of the traditional culture, which includes a longer period of time for some pathogens to report positive and non-comprehensive coverage of all the microorganisms. As a previous study described, whole metagenome shotgun sequencing may be used as a diagnostic procedure to strengthen the diagnosis of IE and to obtain draft genomic sequence of the pathogen and typing information [9]. In our study, the earliest time to report possible detection of *Neisseria* sp. is 1 h, which further illustrated the advantages of the nanopore sequencing in accelerating the diagnostic speed. Currently, new technical advances such as nanopore sequencing have already allowed another step forward of sequencing methods. In our department, we tracked carbapenem-producing *Klebsiella pneumoniae* outbreak in an intensive care unit by Illumina short read and Nanopore long-read sequencing [10]. In comparison to the previous generation sequencing technologies, the new sequencing technologies provide two important advantages: the ability to sequence single molecules, thus avoiding one of the main sources of bias introduced during library preparation, the PCR amplification step, and, most importantly, the increased read length of their output [11].

Most patients with non-HACEK Gram-negative bacteria like *N. macacae* infective endocarditis require extended antibiotic therapy and early surgical evaluation [12]. Optimal antibiotic regimen and duration for *N. macacae*-associated endocarditis are not defined. The prolonged antibiotic therapy for 6 weeks is reasonable [12].

## Conclusion

This is, to our knowledge, the first report of a survival *N. macacae* infective endocarditis case and the first explorative usage of nanopore sequencing in the infectious endocarditis in the world.
